# Hochu-ekki-to Treatment Improves Reproductive and Immune Modulation in the Stress-Induced Rat Model of Polycystic Ovarian Syndrome

**DOI:** 10.3390/molecules22060978

**Published:** 2017-06-13

**Authors:** Eunkuk Park, Chun Whan Choi, Soo Jeong Kim, Yong-In Kim, Samkee Sin, Jong-Phil Chu, Jun Young Heo

**Affiliations:** 1 Department of Medical Zoology, College of Medicine, Kyung Hee University, Seoul 130701, Korea; jude0815@hotmail.com (E.P.); cjp@khu.ac.kr (J.-P.C.); 2 Department of Medical Genetics, College of Medicine, Ajou University School of Medicine, Suwon 443721, Korea; 3 Bio-Center, Gyeonggido Business and Science Accelerator, Suwon 16229, Korea; cwchoi78@gmail.com; 4 Department of Biochemistry, Chungnam National University School of Medicine, Daejeon 301747, Korea; aaron0506@naver.com; 5 Department of Medical Science, Chungnam National University School of Medicine, Daejeon 301747, Korea; 6 International Biological Material Research Center, Korea Research Institute of Bioscience and Biotechnology, Daejeon 34141, Korea; yikim@kribb.re.kr; 7 Institute of Korea Food and Drug Resource, Seoul 06622, Korea; samkee3@hanmail.net; 8 Brain Research Institute, Chungnam National University School of Medicine, Daejeon 301747, Korea

**Keywords:** Hochu-ekki-to, immune modulation, polycystic ovarian syndrome

## Abstract

The traditional herbal medicine, Hochu-ekki-to, has been shown to have preventive effects on viral infection and stress. This study aimed to evaluate the clinical effects of Hochu-ekki-to on two stress-related rat models of polycystic ovarian syndrome. Female Sprague-Dawley rats were divided into control and treatment groups, the latter of which were subjected to stress induced by exposure to adrenocorticotropic hormone (ACTH) or cold temperatures. After these stress inductions, rats were orally treated with dissolved Hochu-ekki-to once per day for 7 days. Rats subjected to the two different stressors exhibited upregulation of steroid hormone receptors (in ovaries) and reproductive hormones (in blood), and consequent stimulation of abnormal follicle development accompanied by elevation of Hsp 90 expression (in ovaries). Treatment with Hochu-ekki-to for 7 days after stress induction increased immune functions, reduced the stress-induced activation of Hsp 90, and normalized the levels of the tested steroid hormone receptors and reproductive hormones. Our findings suggest that stress stimulations may promote the activation of Hsp 90 via the dysregulation of steroid hormone receptors and reproductive hormones, but that post-stress treatment with Hochu-ekki-to improves reproductive and immune functions in the ovaries of stressed rats.

## 1. Introduction

Polycystic ovarian syndrome (PCOS) is the most common endocrine disorder, affecting approximately 5–10% of infertile women of reproductive age. The causative factors of PCOS are correlated with hormone imbalances similar to those normally seen during adolescence; the basic problems of this disease are weight gain, interruption of regular ovulation and menstruation, hirsutism, excessive androgenic activities, and hyperinsulinemia [1]. During PCOS, the ovaries produce excessive amounts of androgens, which can interfere with follicle stimulating hormone (FSH) and trigger the abnormal development of cystic follicles [2]. Polycystic follicles build up in the enlarged ovaries, resulting in irregular or missed ovulation. Although many studies have associated PCOS with an array of metabolic, neuroendocrine, and adrenal abnormalities, the pathogenesis of the polycystic ovary is still unclear.

It has been demonstrated that Japanese herbal medicines such as Hochu-ekki-to may reduce the stress hormones associated with immune functions [3,4]. As an herbal medicine, Hochu-ekki-to has been reported to promote certain biological activities, including the activities of NK cells, mitomycin, and macrophages [5,6]. Currently, this herbal formula is being used in research to treat patients who are resistant to conventional forms of antibiotics [7]. In addition, Hochu-ekki-to has been reported to help relieve stress, decrease fatigue, and suppress carbon tetrachloride-induced hepatotoxicity [8,9,10]. Despite the reported ability of Hochu-ekki-to to inhibit the processes of stress and inflammation, however, no previous study has examined the effect of Hochu-ekki-to on ovarian parameters in rats subjected to stress. Here, we evaluated Hochu-ekki-to as an alternative clinical treatment in two different stress-induced rat models of PCOS: Animals treated with adrenocorticotropic hormone (ACTH) and those exposed to cold stress.

## 2. Results

### 2.1. Effect of Hochu-ekki-to on the mRNA Expression Levels of NGFR and Steroid Hormone Receptors in the Ovaries of Stress-Exposed Rats

**Figure 1:**
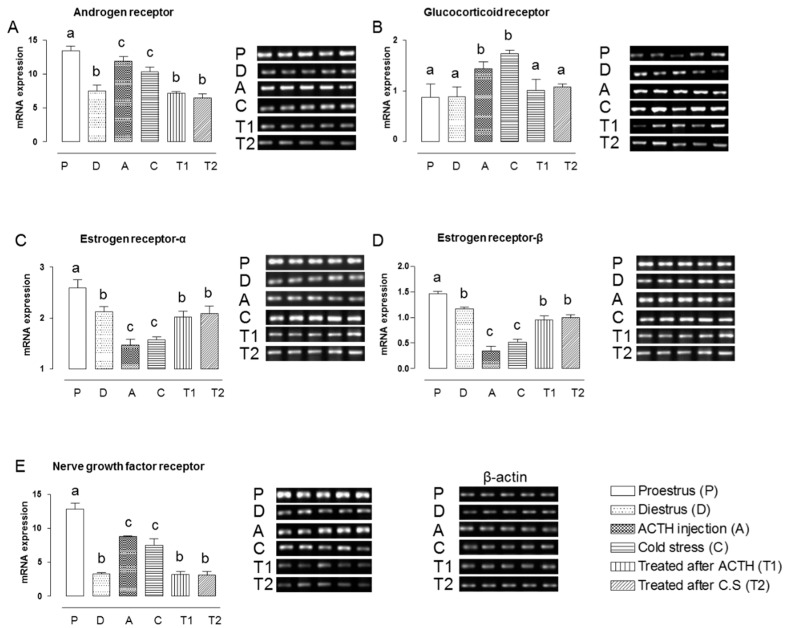
Modulation of steroid hormone receptor in PCOS rat model by administration of Hochu-ekki-to. Mean mRNA expression levels of AR (**A**); ER-α (**B**); ER-β (**C**); NGFR (**D**); and GR (**E**) in the ovaries of the diestrous (D) and proestrous (P) control groups, the ACTH injection group (A), the cold-stress group (CS), and the groups treated with Hochu-ekki-to following ACTH injection (T1) and cold stress (T2). We used the β-actin as an internal control for normalization of mRNA expressions. Means labeled with different letters are significantly different from each other (*p* < 0.05, *n* = 5 in each groups).

First, we tried to analyze the morphology of ovary in ACTH and cold stress induced PCOS rat model. Ovaries from the control group observed various follicles such as primordial, primary, growing secondary and pre-ovulatory tertiary follicles. In contrast, ACTH and cold stress induced PCOS ovaries exhibited ovarian follicle atresia, cortical thickening, low number of corpus luteum and capsular follicular cysts. However, ovarian morphology from the groups treated with Hochu-ekki-to recovered the number of cystic follicles, compared to ACTH and cold stress treated groups (Supplementary Figures S1 and S2). The interaction between NGF and NGFR regulates reproductive functions via both autocrine and paracrine mechanisms in the reproductive tract. Steroid hormone receptors are also involved in regulating reproductive functions [11]. To assess the effect of Hochu-ekki-to on steroid hormone receptors, we used RT-PCR to measure the mRNA expression levels of various receptors in the ovary tissues of stress-exposed rats treated with or without Hochu-ekki-to for 7 days. Among the control (non-stressed) rats, the expression levels of AR, NGFR, ER-α, and ER-β, but not GR, were higher in the proestrous group than in the diestrous group (Figure 1). Both stress exposures upregulated the mRNA expression levels of AR, NGFR and GR and downregulated those of ER-α and ER-β relative to both control groups (*p* > 0.05 for all comparisons); the mRNA expression levels of AR, ER-α, ER-β, NGFR, and GR in the ovary did not differ between the ACTH injection and cold-stress groups (*p* > 0.05, Figure 1D,E). Notably, Hochu-ekki-to treatment after stress exposure restored the mRNA expression levels of the tested steroid hormone receptors and NGFR to control levels (Figure 1).

### 2.2. Effect of Hochu-ekki-to on the mRNA Expression Levels of IL-2, IL-4, and IFN-γ in Stress-Exposed Rats

**Figure 2:**
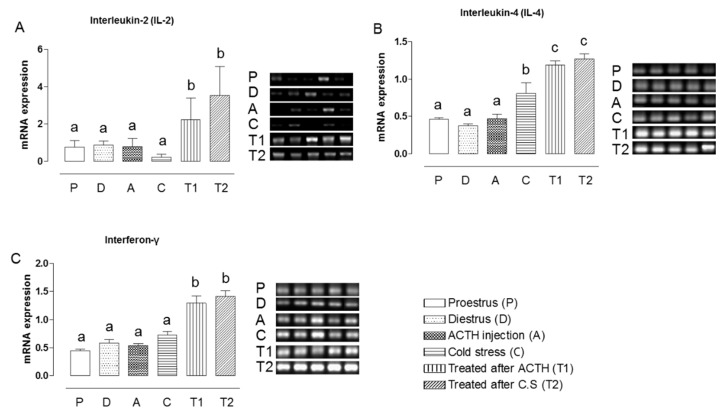
Induction of cytokine expression in PCOS rat model by administration of Hochu-ekki-to. Mean mRNA expression levels of IL-2 (**A**); IL-4 (**B**); and IFN-γ (**C**) in the ovaries of the diestrous (**D**) and proestrous (P) control groups, the ACTH injection group (A), the cold-stress group (CS), and the groups treated with Hochu-ekki-to following ACTH injection (T1) and cold stress (T2). We used the β-actin as an internal control. Means labeled with different letters are significantly different from each other (*p* < 0.05, *n* = 5 in each groups).

Since stress disturbs the immune system and Hochu-ekki-to was reported to improve immune functions in mice by activating the cytokines, IL-2, IL-4, and IFN-γ [12], we examined whether Hochu-ekki-to could improve immune function in our stress-exposed rats. The mRNA expression levels of IL-2, IL-4, and IFN-γ in the ovary presented similar patterns across all groups before Hochu-ekki-to treatment (Figure 2). However, the mRNA expression levels of IL-2, IL-4, and IFN-γ were significantly enhanced by Hochu-ekki-to treatment of the ACTH-injected and cold-stressed rats (*p* < 0.05, Figure 2).

### 2.3. Effect of Hochu-ekki-to on Hsp 90 and MAP2K2 Expression in the Ovaries of Stress-Exposed Rats

**Figure 3:**
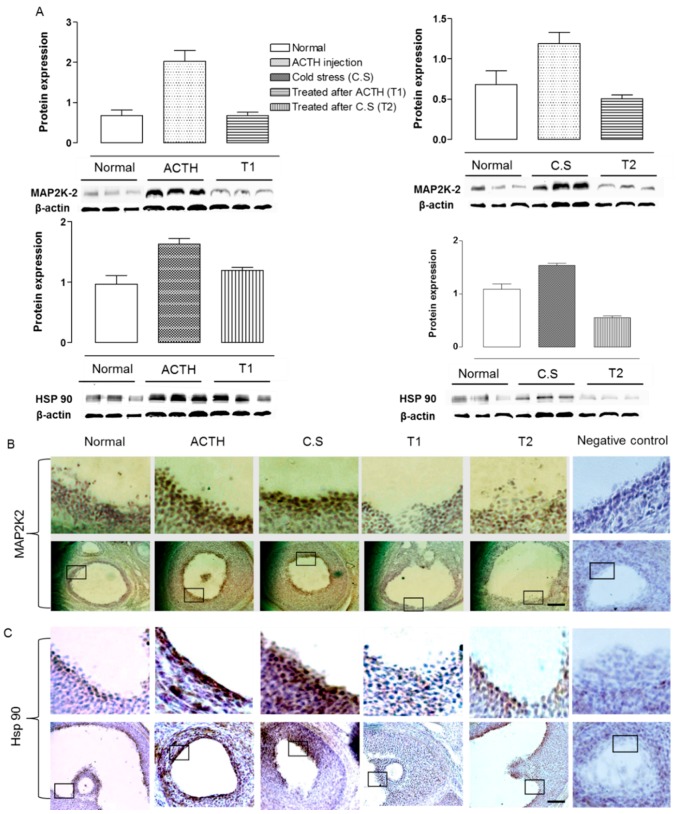
Involvement of MAP2K2 and Hsp90 for Hochu-ekki-to signaling in PCOS rat model. Mean protein expression levels of MAP2K2 and Hsp 90 in the normal diestrous group (Normal), ACTH injection group (ACTH), cold-stress group (C.S), Hochu-ekki-to-treated ACTH injection group (T1) and Hochu-ekki-to-treated cold-stress group (T2) (**A**). Sections of ovaries from normal, ACTH, S.C, T1 and T2 were immunostained for MAP2K2 (**B**) and Hsp 90 (**C**). Negative control was stained without primary antibody. Means labeled with different letters are significantly different from each other (*p* < 0.05, *n* = 5 in each groups). Scale bar 50 μm.

Steroid hormone receptors crucially depend on interactions with Hsp 90, and mitogen-activated protein kinase kinase 2 (MAP2K2) is upregulated during the response to stress. Indeed, the protein expression levels and immunohistochemical analysis of Hsp 90 and MAP2K2 in the ovary were significantly increased in both ACTH-injected and cold-exposed rats, compared to controls (*p* < 0.05, Figure 3). However, Hochu-ekki-to treatment for 7 days following the application of stress reduced the protein expression levels of Hsp 90 (Figure 3).

### 2.4. Hormone Assay

**Figure 4:**
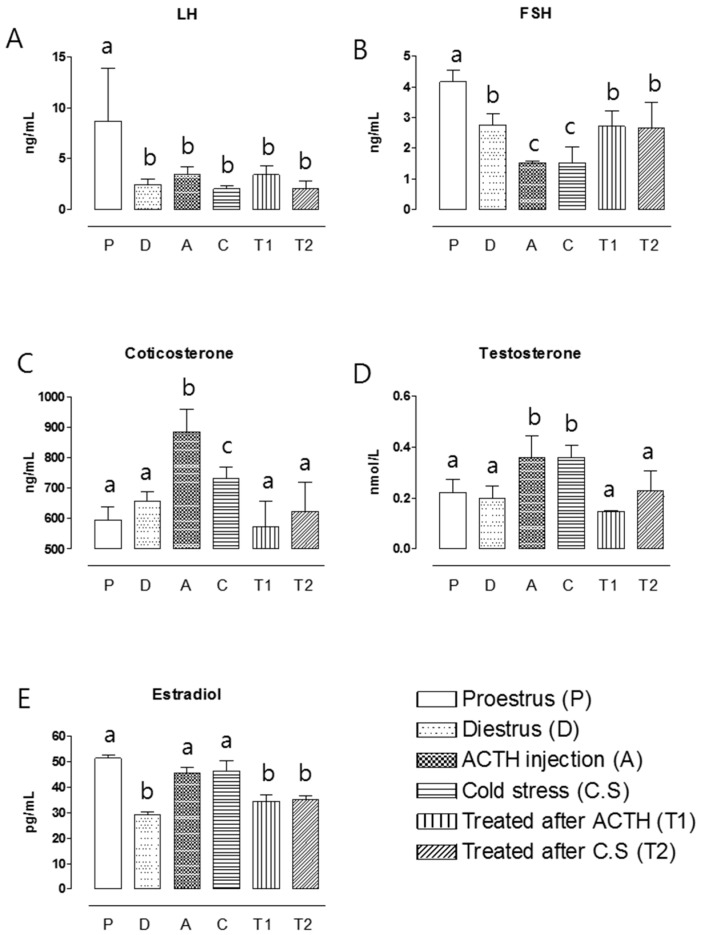
Measurement of intravascular steroid hormone level in PCOS rat model with/without Hochu-ekki-to treatment. Serum LH (**A**); FSH (**B**); corticosterone (**C**); testosterone (**D**) and estradiol (**E**) concentrations measured in the ovaries of rats from the diestrous control (D), proestrous control (P), ACTH injection group (A), cold-stress (CS), Hochu-ekki-to-treated ACTH injection (T1), and Hochu-ekki-to-treated cold-stress (T2) groups. Means labeled with different letters are significantly different from each other (*p* < 0.05, *n* = 5 in each groups).

To verify the stress-induced alterations of reproductive function, blood samples were assessed for the concentrations of various hormones. In control rats, higher concentrations of FSH, LH, and estradiol were observed in the pro-estrous group. The ACTH injection and cold-stress groups both showed significant reductions (*p* < 0.05 for all comparisons) in FSH (Figure 4A), testosterone, and corticosterone (Figure 4D,E). The post-stress administration of Hochu-ekki-to for 7 days further inhibited the testosterone and corticosterone levels, but increased the FSH level, compared to the stress-induced groups. The concentrations of FSH, LH, estradiol, corticosterone, and testosterone did not differ between the two Hochu-ekki-to-treated groups (Figure 4). There was no significant difference in the LH and estradiol concentrations of the diestrous, stress-induced, and Hochu-ekki-to treated groups; the proestrous group, in contrast, showed significantly higher LH and estradiol concentrations than all other groups (Figure 4). These results suggest that the administration of Hochu-ekki-to for 7 days could rescue stress-induced alterations of reproductive hormones.

## 3. Discussion

This study examined the efficacy of Hochu-ekki-to in two different stress-induced rat models of PCOS, namely animals treated with ACTH injection and cold stress. Hochu-ekki-to is a traditional Japanese herbal medicine that is composed of 10 species of medicinal plants. Hochu-ekki-to has been reported to reduce complaints of general fatigue caused by common colds, and to improve severe weakness. Many studies have shown that it has various immunoactive effects, such as increasing immunity in elderly persons [6], augmenting natural killer cell activity [13], and increasing the production of cytokines [14]. In this study, the ACTH injection and cold-stress groups both showed significant increases in their mRNA expression levels of glucocorticoid receptor (GR), IL-2, IL-4, and IFN-γ and concentrations of corticosterone, compared to the corresponding control groups (Figure 1, Figure 2 and Figure 4), but these effects were rescued by administration of Hochu-ekki-to for 7 days.

Gonadotrophin-releasing hormone (GnRH) is a key sexual-behavior hormone that acts between the neural and endocrine systems [15]. GnRH from the hypothalamus stimulates the anterior pituitary to secrete FSH and LH; this supports the production of steroid hormones, which promote folliculogenesis and the concomitant synthesis of estradiol [16]. Steroid hormone receptors, such as GR, AR, ER-α, and ER-β, critically regulate the actions of gonadotrophin in association with the function of the HPG axis [11]. In the presence of stress, however, ovulation can fail due to inappropriately programmed activity of the hypothalamus. Studies have shown that stress suppresses LH secretion in the absence of estrogen; for example, immobilization and foot-shock stress were found to strongly inhibit LH secretion in ovariectomized rats [17,18]. In the present study, the mRNA expression levels of AR, ER-α, and ER-β and the concentrations of LH, FSH, and estradiol were significantly higher in the proestrous group compared to the other groups. In addition, ACTH injection and cold-stress groups increased the mRNA expression levels of AR and plasma concentrations of testosterone, but decreased those of ER-α, and ER-β and concentration of FSH. Following Hochu-ekki-to treatment, however, there was no difference between the diestrous control and treated groups with respect to the mRNA levels of AR, ER-α, and ER-β in the ovary or the plasma concentrations FSH, LH, estradiol, and testosterone. These results suggested that the administration of Hochu-ekki-to for 7 days could rescue these stress-induced effects.

HSP 90 is a molecular chaperone that is upregulated in response to elevated temperature-induced stress [19,20]. One of the main functions of Hsp 90 is to interact with steroid receptors. In the absence of the hormone cortisol, the GR (which is involved in cell-mediated immunity) resides in the cytosol in complex with a variety of proteins, including Hsp 90, to which it binds directly [21]. Hsp 90 also binds several other steroid receptors, including aldosterone, androgen, estrogen, and progesterone [11]. In addition, mitogen-activated protein kinase (MAPK), which contributes to regulating cell differentiation and specifying sensory neuron subtypes, also appears to mediate the functions of nerve growth factor (NGF) via a signaling complex that includes Hsp 90 [22]. Although MAPK largely acts as non-nuclear oncogenes, they are additionally involved in the cellular response to growth factors, such as brain-derived neurotrophic factor (BDNF) and NGF. It has been reported that excessive activation of MAPK by oxidative stress and hyperinsulinemia may promote the incidence of PCOS [23]. Human mitogen-activated protein kinase kinase 2 (MAP2K2), which belongs to the MAP kinase kinase family, is known to play a critical role in mitogen growth factor signal transduction [22]. Here, we found that the mRNA expression levels of NGFR, and protein expressions of both MAP2K2 and Hsp 90 were significantly higher in ACTH injection and cold-stress groups, compared to diestrous control groups, but they were rescued to control levels by Hochu-ekki-to treatment. This suggests that Hochu-ekki-to may inhibit the stress-induced productions of NGFR, MAP2K2 and Hsp 90. The present study found that a 7-day treatment of Hochu-ekki-to after the induction of stress by ACTH injection or cold exposure improved the ovarian reproductive and immune functions associated with POCS in a rat model.

## 4. Materials and Methods

### 4.1. Animals and Treatments

Adult female Wistar rats weighing 180–200 g (7–8 weeks of age) were obtained from Dae Han BioLINK (Emsung, Korea) and kept in a central animal care facility under a 12-h light/dark cycle (lights on at 06:00 h) and controlled temperature (24 ± 0.5 °C). Food and water were provided ad libitum. All mouse experiments were performed in the animal facility according to institutional guidelines (SOP; standard operating procedure), and the experimental protocols were approved by the institutional review board of Chungnam National University (CNU-00356).

Thirty rats were divided into six groups of five rats each: two control groups, one proestrous and one diestrous; groups subjected to ACTH injection with and without subsequent administration of Hochu-ekki-to; and groups subjected to cold stress with and without subsequent administration of Hochu-ekki-to. The controls were divided into proestrous (*n* = 5) and diestrous (*n* = 5) groups due to the significant alteration seen in the reproductive hormonal pattern during the proestrous period. Therefore, we used the diestrous group as a control group. Stressed rats elicited regular estrous cycles and we used only diestrous rats. The animals in the ACTH treatment groups (*n* = 10) were subcutaneously injected with a dose equivalent to 20 UI/kg (200 μg/kg) of ACTH (ProSpec-Tany TechnoGene, Rehovot, Israel) every 24 h for 18 days. The animals in the cold-stress treatment group (*n* = 10) were placed at 4 °C for 3 h per day (from 11:00 to 14:00) for 3 weeks. The ACTH (*n* = 5) or cold-treated (*n* = 5) rats were orally treated with dissolved Hochu-ekki-to (500 mg/kg) once a day for 7 days through a feeding needle inserted down the throat. The remaining ACTH (*n* = 5) or cold-treated rats (*n* = 5) were orally treated with distilled water.

On the last day of each treatment, rats were anesthetized and blood samples were collected from the left ventricle (*n* = 5 in each groups). The blood concentrations of FSH, LH, estradiol, testosterone, and corticosterone were measured in all groups. Samples of ovary were removed, ovulation was assessed, and the rats in two control groups were divided into one proestrous and one diestrous groups. Each right ovary was used for histological analysis, while the left ovary was divided into two parts and analyzed for the mRNA and protein expression levels of various factors. We identified the folliculogenesis in the ovary through ACTH and cold stress which represents phenotype of polycystic ovarian syndrome (Supplementary Figures S1 and S2). All experiment was performed at least different three times.

### 4.2. Tissue Sampling

Animals were euthanized immediately before tissue collection. Each animal was placed into the CO_2_ chamber to initiate the flow of CO_2_. Tissues were serially sectioned using a stainless steel blade, and then immediately frozen in dry ice and stored at −80 °C.

### 4.3. RNA Extraction and Reverse Transcription Polymerase Chain Reaction (RT-PCR)

RT-PCR was conducted on samples from four animals of each group. Total RNA was isolated using the TRIzol reagent (Thermo Fisher Scientific, Waltham, MA, USA) according to the manufacturer’s instructions. RNA concentrations were quantified with a Nano-Drop (Thermo Fisher Scientific). The specific primers were as follows: androgen receptor AR F (5′-agctcaccaagctcctggat-3′), AR R (5′-aagggaacaaggtgggtttg-3′), estrogen receptor-α ER-α F (5′-gaccatgacccttcacacca-3′), ER-α R (5′-gttgtccacgtacacctcgc-3′), ER-β F (5′-gtgaaggccatgatcctcct-3′), ER-β R (5′-agtcggactgactgctgctg -3′), nerve growth factor receptor NGFR F (5′-aagagatccctggtcgatgg-3′), NGFR R (5′-gcagccaagatggagcaata-3′), glucocorticoid receptor GR F (5′-attttcacatctcacccgca-3′), GR R (5′-agtagccctttccctttccc-3′), interleukin-2 IL-2 F (5′-cacttggaagacgctggaaa-3′), IL-2 R (5′-cccttggggcttacaaaaag-3′), interleukin-4 IL-4 F (5′-cggtatccacggatgtaacg-3′), IL-4 R (5′-ctccgtggtgttccttgttg-3′), interferon-γ INF-γ F (5′-cccacagatccagcacaaag-3′), INF-γ R (5′-ttggcacactctctacccca-3′), beta-actin β-actin F (5′-ctaggcaccagggtgtgatg-3′), β-actin R (5′-ggggtacttcagggtcagga-3′).

RT-PCR was performed using an Access RT-PCR System (Promega, Madison, WI, USA) and a Genius thermal cycler (Bio-Rad, Hercules, CA, USA) according to the manufacturer’s instructions. The RT-PCR products (5 μL each) were resolved at 135 V for 30 min on a 1.2% agarose gel (Bio-Rad, Hercules, CA, USA) and visualized by ethidium bromide staining (Sigma-Aldrich, St. Louis, MO, USA) in TBE buffer (In Vitro Technologies Ltd., Biotek, Seoul, Korea). The DNA in appropriately-sized bands was quantified under UV light using a Bio-Rad Chemi-Doc system (Bio-Rad). The values obtained for β-actin were used to correct for differences in the amount of cDNA in each sample. 

### 4.4. Western Blotting Analysis

Total proteins were isolated from frozen ovary samples using lysis buffer (Bio-Rad) according to the manufacturer’s instructions. Briefly, the tissues were washed twice with cold PBS and lysed with RIPA lysis buffer for 25 min. The lysates were sonicated three times at 20-second intervals, aliquotted, and stored at −20 °C. The protein concentrations were determined using a DC protein assay kit (Bio-Rad), and equal amounts of protein were subjected to 15% SDS-PAGE and transferred to nitrocellulose membranes. The membranes were incubated overnight at 4 °C with primary antibodies against Hsp 90 (1:1000; Assay Designs, Stressgen, San Diego, CA, USA) or β-actin (1:2000; R&D Systems, Minnealpolis, MN, USA). The membranes were washed with PBS-Tween (0.05%), and then incubated with goat anti-mouse or anti-rabbit IgG HRP-conjugated secondary antibodies for 90 min at room temperature. Antibody-bound proteins were visualized using an ECL western blotting analysis system (Amersham Pharmacia Biotech UK Limited, Buckinghamshire, UK) and assessed with a Chemi-Doc system (Bio-Rad). The membranes were first probed with the Hsp 90 antibody, and then subsequently stripped and reprobed with β-actin antibody as an internal control and to confirm equal loading.

### 4.5. Immunohistochemistry

For the immunohistochemical detection of Hsp 90, free-floating sections were pre-incubated for 30 min in a 1% solution of H_2_O_2_, and then incubated overnight at 25 °C in 0.3% Triton X-100, 0.5 mg/mL bovine serum albumin, and a polyclonal antibody against Hsp 90 (1:100 dilution; Assay Designs, Stressgen). Each section was then incubated for 120 min with the appropriate secondary antibody (1:200; Vector, Burlingame, CA, USA) and treated with an avidin-biotin-peroxidase complex (1:100; Vector) for 1 h at room temperature. Peroxidase activity was visualized by incubating the section with 0.02% DAB and 0.01% H_2_O_2_ in 0.5 M Tris-buffered saline (pH 7.6). After several rinses, the sections were mounted on gelatin-coated slides, counterstained with Mayer’s hematoxylin, dehydrated, and cover-slipped using Histomount medium (Invitrogen, Camarillo, CA, USA).

### 4.6. Blood Samples

Blood samples (approximately 5 mL) were collected into heparinized syringes from the left ventricles of anesthetized rats. Plasma was separated by centrifugation at 1200× *g* for 15 min at 4 °C and stored at −20 °C until use. Each sample was assayed for LH and FSH by enzyme-linked immunosorbent assay (ELISA), and estradiol, testosterone and corticosterone levels were assessed by radioimmunoassays (RIAs). 

### 4.7. ELISA of FSH and LH

The serum concentrations of LH and FSH were measured by sensitive and specific competition ELISA (Merck Millipore, Temecula, CA, USA), following the manufacturer’s instructions. The rat LH ELISA was performed using rLH-I-9-coated plates, an antiserum against rLH, and a peroxidase-labeled antibody against rabbit IgG. Using rLH-RP-3 as a standard, the level of rat LH was determined by the binding of the anti-LH antibody to the rLH-I-9 coated plates. The sensitivity of the assay was 0.8 ng/mL. Similarly, the rat FSH-ELISA was performed using rFSH-I-8 coated plates, an antiserum against rFSH, and a peroxidase-labeled antibody against rabbit IgG. Using rFSH-RP-3 as a standard, the level of FSH was determined by the binding of the anti-FSH antibody to the rFSH-I-8 coated plates. The sensitivity of this assay was 1.25 ng/mL. 

### 4.8. RIAs for Testosterone, Corticosterone and Estradiol 

For measurement of serum testosterone (expressed as ng/dL), duplicate 50-μL samples were assayed using a Coat-A-Count Total Testosterone kit (Siemens Medical Solutions Diagnostics, Malvern, PA, USA), following the manufacturer’s instructions. The assay sensitivity was 0.14 nmol/L. All samples were measured in a single assay; the intra-assay coefficients of variation for the two assays were 6.4%, 5.9%, and 7.3% for the low (264 ng/dL), medium (594 ng/dL), and high (1300 ng/dL) solutions, respectively. For measurement of serum corticosterone concentrations (expressed as ng/mL), duplicate 50-μL aliquots were assayed using a Coat-A-Count Total Corticosterone kit (Siemens Medical Solutions Diagnostics), which applies solid-phase RIA using a testosterone-specific antibody immobilized to the wall of a polypropylene tube. The assay sensitivity was 5.7 ng/mL. The inter-assay coefficients of variation for the two assays were 12.2%, 4.3%, and 4.0% for the low (24.6 ng/mL), medium (154 ng/mL), and high (427 ng/mL) solutions, respectively. For measurement of serum estradiol concentrations (expressed as pg/mL), duplicate 100-μL aliquots were assayed using a Coat-A-Count Total estradiol kit (Siemens Medical Solutions Diagnostics), which uses antibody-coated tubes. The analytical sensitivity was basic procedure for 8 pg/mL (29 pmol/L). The inter-assay coefficients of variation for the two assays were 7.4%, 5.5% and 4.2% for the low (90 pg/mL), medium (262 pg/mL), and high (1025 ng/mL) solutions, respectively. 

### 4.9. Statistical Analyses

SPSS 11.0 for Windows (SPSS Inc., Chicago, IL, USA) was used to perform the statistical tests. The statistical significance of differences was assessed by one-way ANOVA, followed by Duncan’s multiple range test for multiple comparisons. *P* < 0.05 was considered significant. The results are expressed as the mean ± SEM.

## Supplementary Materials

Supplementary materials are available online.

## References

[1] Haq F., Rizvi J. (2008). Infertility and polycystic ovarian syndrome: A study of association between body mass index and intrafamily marriages. Gynecol. Obstet. Investig..

[2] Norman R.J., Dewailly D., Legro R.S., Hickey T.E. (2007). Polycystic ovary syndrome. Lancet.

[3] Kimura M., Sasada T., Kanai M., Kawai Y., Yoshida Y., Hayashi E., Iwata S., Takabayashi A. (2008). Preventive effect of a traditional herbal medicine, hochu-ekki-to, on immunosuppression induced by surgical stress. Surg. Today.

[4] Ohgitani E., Kita M., Mazda O., Imanishi J. (2014). Combined administration of oseltamivir and hochu-ekki-to (tj-41) dramatically decreases the viral load in lungs of senescence-accelerated mice during influenza virus infection. Arch. Virol..

[5] Cho S., Hong T., Kaneko A., Yoshino G., Sato N., Kikuchi K., Aikawa Y., Yasuno F., Inoue K., Cyong J.C. (2004). Evaluation of immunological effects of hochu-ekki-to (tj-41) prophylactic administration in mice. Am. J. Chin. Med..

[6] Kuroiwa A., Liou S., Yan H., Eshita A., Naitoh S., Nagayama A. (2004). Effect of a traditional Japanese herbal medicine, hochu-ekki-to (bu-zhong-yi-qi tang), on immunity in elderly persons. Int. Immunopharmacol..

[7] Yan X., Kita M., Minami M., Yamamoto T., Kuriyama H., Ohno T., Iwakura Y., Imanishi J. (2002). Antibacterial effect of kampo herbal formulation hochu-ekki-to (bu-zhong-yi-qi-tang) on helicobacter pylori infection in mice. Microbiol. Immunol..

[8] Kobayashi H., Mizuno N., Teramae H., Kutsuna H., Ueoku S., Onoyama J., Yamanaka K., Fujita N., Ishii M. (2004). The effects of hochu-ekki-to in patients with atopic dermatitis resistant to conventional treatment. Int. J. Tissue React..

[9] Wang X.Q., Takahashi T., Zhu S.J., Moriya J., Saegusa S., Yamakawa J., Kusaka K., Itoh T., Kanda T. (2004). Effect of hochu-ekki-to (tj-41), a Japanese herbal medicine, on daily activity in a murine model of chronic fatigue syndrome. eCAM.

[10] Yoshioka H., Fukaya S., Onosaka S., Nonogaki T., Nagatsu A. (2016). Kampo formula “hochu-ekki-to” suppressed carbon tetrachloride-induced hepatotoxicity in mice. Environ. Health Prev. Med..

[11] Beato M., Klug J. (2000). Steroid hormone receptors: An update. Hum. Reprod. Update.

[12] Nakada T., Watanabe K., Matsumoto T., Santa K., Triizuka K., Hanawa T. (2002). Effect of orally administered hochu-ekki-to, a Japanese herbal medicine, on contact hypersensitivity caused by repeated application of antigen. Int. Immunopharmacol..

[13] Utsuyama M., Seidlar H., Kitagawa M., Hirokawa K. (2001). Immunological restoration and anti-tumor effect by Japanese herbal medicine in aged mice. Mech. Ageing Dev..

[14] Yang S.H., Kao T.I., Chiang B.L., Chen H.Y., Chen K.H., Chen J.L. (2015). Immune-modulatory effects of bu-zhong-yi-qi-tang in ovalbumin-induced murine model of allergic asthma. PLoS ONE.

[15] Engmann L., DiLuigi A., Schmidt D., Nulsen J., Maier D., Benadiva C. (2008). The use of gonadotropin-releasing hormone (GnRH) agonist to induce oocyte maturation after cotreatment with GnRH antagonist in high-risk patients undergoing in vitro fertilization prevents the risk of ovarian hyperstimulation syndrome: A prospective randomized controlled study. Fertil. Steril..

[16] Carriere P.D., Brawer J.R., Farookhi R. (1991). Alterations in gonadotropin-releasing hormone-dependent gonadotropin secretion in rats with polycystic ovaries. Biol. Reprod..

[17] Hemmings R., Farookhi R., Brawer J.R. (1983). Pituitary and ovarian responses to luteinizing hormone releasing hormone in a rat with polycystic ovaries. Biol. Reprod..

[18] Dobson H., Ribadu A.Y., Noble K.M., Tebble J.E., Ward W.R. (2000). Ultrasonography and hormone profiles of adrenocorticotrophic hormone (ACTH)-induced persistent ovarian follicles (cysts) in cattle. J. Reprod. Fertil..

[19] Csermely P., Schnaider T., Soti C., Prohaszka Z., Nardai G. (1998). The 90-kda molecular chaperone family: Structure, function, and clinical applications. A comprehensive review. Pharmacol. Ther..

[20] Park E., Cockrem J.F., Han K.H., Kim D.H., Jung M.H., Chu J.P. (2012). Stress-induced activation of ovarian heat shock protein 90 in a rat model of polycystic ovary syndrome. J. Obstet. Gynaecol. Res..

[21] Wadekar S.A., Li D., Periyasamy S., Sanchez E.R. (2001). Inhibition of heat shock transcription factor by GR. Mol. Endocrinol..

[22] Jaiswal R.K., Weissinger E., Kolch W., Landreth G.E. (1996). Nerve growth factor-mediated activation of the mitogen-activated protein (map) kinase cascade involves a signaling complex containing b-raf and hsp90. J. Boil. Chem..

[23] Lan C.W., Chen M.J., Tai K.Y., Yu D.C., Yang Y.C., Jan P.S., Yang Y.S., Chen H.F., Ho H.N. (2015). Functional microarray analysis of differentially expressed genes in granulosa cells from women with polycystic ovary syndrome related to mapk/erk signaling. Sci. Rep..

