# Supplementary material for: Hochu-ekki-to Treatment Improves Reproductive and Immune Modulation in the Stress-Induced Rat Model of Polycystic Ovarian Syndrome
Source: Molecules. 2017 Jun 13;22(6):978. doi: 10.3390/molecules22060978 (PMC6152635; doi:10.3390/molecules22060978)
Supplement: Supplementary file 1 [file molecules-22-00978-s001.pdf]

Hochu-ekki-to treatment improves reproductive and immune modulation in the stress-induced rat model of polycystic ovarian syndrome

Short title: Hochu-ekki-to and polycystic ovarian syndrome

Eunkuk Park [1,7], Chun Whan Choi [2], Soo Jeong Kim [3,4], Yong-In Kim [5], Samkee Sin [6], Jong-Phil Chu [1] and Jun Young Heo [3,4,8]*

1 Department of Medical Zoology, College of Medicine, Kyung Hee University, Seoul 130701, Korea; jude0815@hotmail.com; depara@khu.ac.kr

2 Bio-Center, Gyeonggido Business and Science Accelerator, Suwon 16229, Korea; cwchoi78@gmail.com

3 Department of Biochemistry, Chungnam National University School of Medicine, Daejeon 301747

4 Department of Medical Science, Chungnam National University School of Medicine, Daejeon 301747, Korea; aaron0506@naver.com

5 International Biological Material Research Center, Korea Research Institute of Bioscience and Biotechnology, Daejeon 34141, Korea; yikim@kribb.re.kr

6 Institute of Korea Food and Drug Resource, Seoul 06622, Korea; samkee3@hanmail.net

7 Department of Medical Genetics, College of Medicine, Ajou University School of Medicine, Suwon 443721 Republic of Korea

8 Brain Research Institute, Chungnam National University School of Medicine, Daejeon 301747, Republic of Korea

Supplementary Figures S1, S2 and Figure Legends

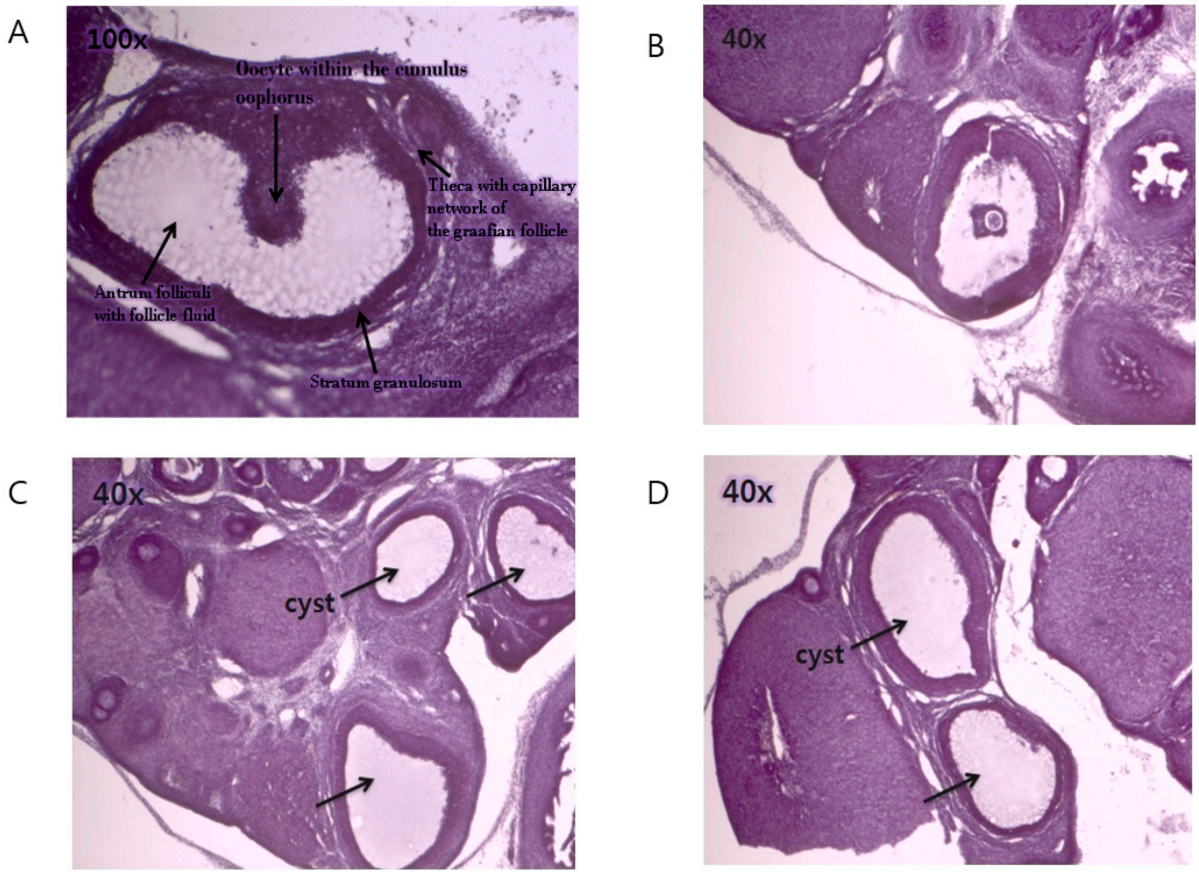

S1 Figure. Ovarian morphology was assessed by hematoxylin-eosin staining. (A and B) The morphologies of dominant follicles before ovulation. (C and D) Ovarian morphologies on estrous of rats subjected to ACTH injection and cold stress

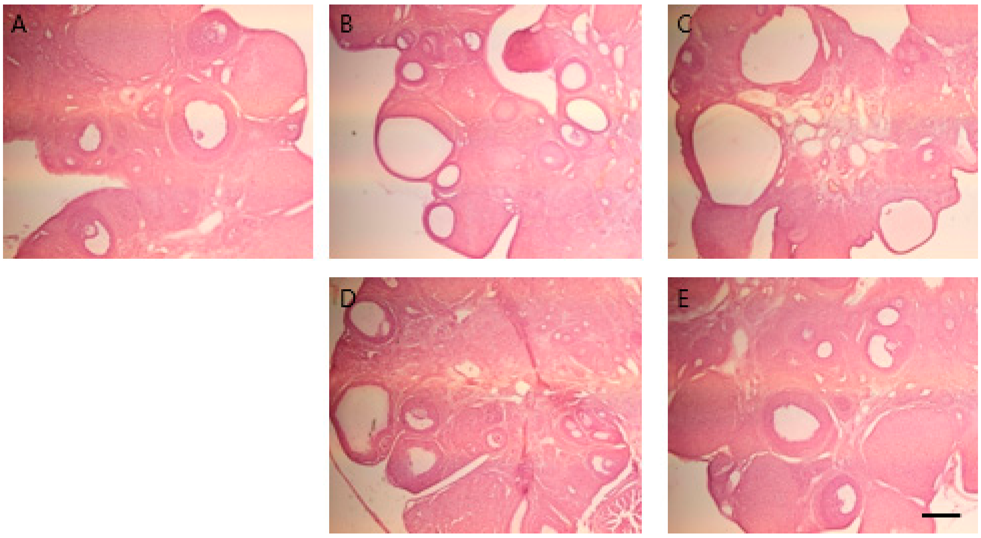

S2 Figure. Ovarian morphology was assessed by hematoxylin-eosin staining of ovary sections from rats of the control group (A), ACTH injection group (B), cold-stress group (C), Hochu-ekki-to-treated ACTH injection group (D), and Hochu-ekki-to-treated cold-stress group (E).
